# Variation in the use of chemotherapy in lung cancer

**DOI:** 10.1038/sj.bjc.6603659

**Published:** 2007-03-06

**Authors:** N Patel, R Adatia, A Mellemgaard, R Jack, H Møller

**Affiliations:** 1 King's College London, Pharmaceutical Science Research Division, Franklin-Wilkins Building, 150 Stamford Street, London SE1 9NH, UK; 2 Department of Oncology, Herlev University Hospital, Herlev, Copenhagen, Denmark; 3 King's College London, Thames Cancer Registry, Capital House, 42 Weston Street, London, SE1 3QD

**Keywords:** chemotherapy, cancer networks, lung cancer, cancer plan, NSCLC, SCLC

## Abstract

Factors influencing the use of chemotherapy for the initial (6 months) treatment of lung cancer in South East England were investigated. The variables explored as possibly influencing the use of chemotherapy were sex, age, the year of diagnosis, the type of lung cancer, the stage, the index of multiple deprivation and the cancer network of residence. *χ*^*2*^ analysis and multivariate logistic regression models were used to examine the effect of each of the variables on the use of chemotherapy. The results showed a highly significant trend in use of chemotherapy over time; the adjusted proportion of patients receiving chemotherapy increasing from 13.6% in 1994 to 29.3% in 2003. However, age, cancer network and type of lung cancer had the strongest influence on the use of chemotherapy. This finding is important when we consider that the NHS Cancer Plan aims at improving inequalities in cancer care in the UK.

Cancer is a major problem in the UK and inequalities in cancer care have received much media interest. In 1995, the UK Department of Health recommended a national framework for cancer services in the UK (DoH, 1995). The NHS Plan (DoH, 2000a) presented the government's strategy for investment and reform across the NHS, and gave cancer services a high priority. From this, the Cancer Plan (DoH, 2000b) was published, which set out a comprehensive strategy for development of cancer care and cancer services. One of the objectives is to reduce the incidence of lung cancer and a number of objectives have been proposed to ensure improved survival from the disease. Smoking, which is the single most common cause of lung cancer and is responsible for the disease occurrence (80–90% cases) (Doll *et al*, 1994; Peto *et al*, 2000), is highest on the agenda with an aim to reduce smoking prevalence from 32% in 1998 to 26% by 2010. The Cancer Plan has also set the target that by 2010, the 5-year survival for all cancers will compare with the highest in Europe (DoH, 2000b).

Lung cancer is a common cause of death in the Western world with 178 000 deaths in the US in 2002 and 38 000 in the UK per year (NICE, 2005). For clinical, therapeutic and biological reasons, lung cancers are frequently classified into small cell (SCLC) and non-small cell (NSCLC). Non-small-cell lung cancer accounts for 80% of all lung cancers and can be further divided into squamous carcinoma, large-cell carcinoma and adenocarcinoma (including bronchioloalveolar). Commonly, these subdivisions are grouped together because approach to the diagnosis, staging, prognosis and treatment are similar (Mason, 2005).

Treatment options for lung cancer include surgery, chemotherapy and radiotherapy and depend on stage and performance status of the patient. For NSCLC, the majority of patients are diagnosed with advanced stage (IIIB and IV) and treatment (nonsurgical) is predominantly palliative. In recent years, adjuvant chemotherapy has been introduced following surgery for stage IB-IIIA. Although chemotherapy has been established as treatment for SCLC since the 1980s (Murray, 1997; Todd, 2005), chemotherapy for NSCLC has only been supported by clinical studies in the past 10 years (Spiro and Silvestre, 2005). Guidelines for treatment of lung cancer have been published by the National Institute of Clinical Excellence (NICE) in 2005 and by the American Society of Clinical Oncology in 1999 with an update in 2003. Both guidelines recommend chemotherapy for advanced stage patients (stage III and IV NSCLC) in good performance.

The aim of this work was to explore variations and trends in the use of chemotherapy as initial treatment in lung cancer. The two main types of lung cancer are distinguished in this study but no distinctions are made between the different types of chemotherapy. We have limited our selection of explanatory variables to those thought most pertinent and routinely collected by the Thames Cancer Registry (TCR).

## MATERIALS AND METHODS

The Thames Cancer Registry is the largest population based cancer registry in Europe. In the period up to 2005, it covered a population of over 14 million people in the South East of England, comprising London, Essex, Sussex, Surrey, Kent and Hertfordshire. The TCR obtains its data from a wide range of sources, including pathology reports and case notes from NHS Trusts and other medical institutions, the Office for National Statistics (ONS), and from other cancer registries. The data for this analysis were extracted from the TCR for 67 312 patients diagnosed with lung cancer between 1994 and 2003, who were not registered by death certificate only. Of these patients, 11 215 received chemotherapy within 6 months of diagnosis. This is the period in which routine cancer registration collects treatment information. Explanatory factors examined were the sex of the patient, age of the patient, year of diagnosis, type of lung cancer, cancer stage, index of multiple deprivation (IMD) and the cancer network in which the patient was resident.

The IMD was assessed on the basis of postcode of residence and classified into quintiles (most affluent ranked as 1, and the least affluent ranked as 5). The IMD is based on certain domains of deprivation, each made of certain indicators to cover all aspects of socio-economic deprivation comprehensively.

Lung cancer type was differentiated as NSCLC, SCLC and NOS (cancer not otherwise specified). The staging of NSCLC was based on the TNM system, which was simplified in this study into stages I to IV as reported in NICE guidelines (2005). SCLC staging classification is defined as limited stage disease and extensive stage disease and is not reported in this study. Patients were assigned to a cancer network of residence based on their postcode at the time of diagnosis. We report both the unadjusted and adjusted proportions of patients who have received chemotherapy within the first 6 months of diagnosis.

STATA (v.8.2) software was used for all statistical calculations. Univariate comparisons were carried out using *χ*^*2*^ analysis. On the basis of the explanatory variables, multivariate logistic regression models were used to examine the likelihood that each of the variables had an effect on the patients subsequently receiving chemotherapy. For each variable, the proportions were adjusted to allow for differences in all of the other factors. *P*-values for trend or for heterogeneity were computed as appropriate.

## RESULTS

Table 1 shows the results of the logistic regression analysis. Both unadjusted and adjusted proportions for the different factors tested for patients receiving chemotherapy are shown. There was a highly significant trend in use of chemotherapy and age. There were a greater proportion of patients receiving chemotherapy in the younger age groups and this trend was not sensitive to statistical adjustment for other factors. The dependence on age was present within individual strata of other variables, for example, cancer network.

A comparison between male and female subjects showed no significant difference between the initiation of chemotherapy (16.7 and 16.6%, respectively). After adjusting for the other factors, male subjects (16.7%) were more likely than female subjects (15.4%) to receive chemotherapy.

There was a significant trend with IMD. A greater proportion of patients in the least deprived group (quintile 1) received chemotherapy. After having adjusted for the other variables, the trend became more pronounced and highly statistically significant.

**Figure 1:**
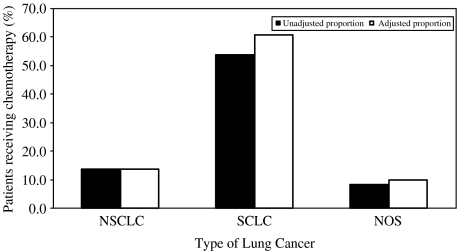
Percentage of patients receiving chemotherapy according to the type of lung cancer. NSCLC denotes non-small-cell lung cancer; SCLC denotes small-cell-lung cancer; NOS denotes cancer not otherwise specified.

Figure 1 shows the percentage of patients receiving chemotherapy based on cancer type. There were a greater proportion of patients with SCLC being treated with chemotherapy.

**Figure 2:**
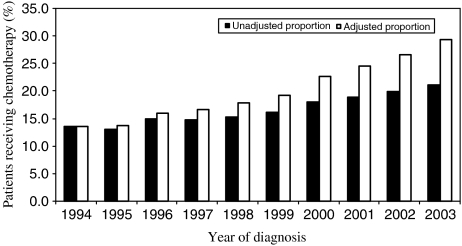
Percentage of patients receiving chemotherapy according to the year of diagnosis.

Figure 2 shows that there was a significantly increasing trend in the proportion of patients receiving chemotherapy over the 10-year period. The proportion of patients receiving chemotherapy increased constantly over time from 13.6% in 1994 to 21.2% in 2003 (unadjusted proportions). In the adjusted model, the gradient became even steeper.

**Figure 3:**
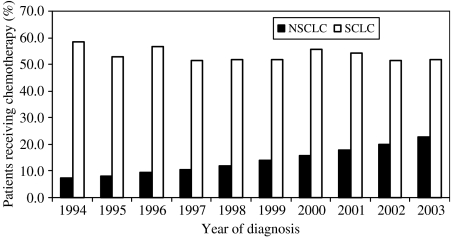
Unadjusted proportions of NSCLC and SCLC patients receiving chemotherapy by year of diagnosis. NSCLC denotes non-small-cell lung cancer; SCLC denotes small-cell-lung cancer

Figure 3 shows that over the 10-year period there was an increase in the proportion of patients receiving chemotherapy who have NSCLC from 7.2% in 1994 to 22.8% in 2003 (unadjusted proportions). The trend does not seem to change over time in chemotherapy use in patients with SCLC (variation between 51.3 and 58.4%).

**Figure 4:**
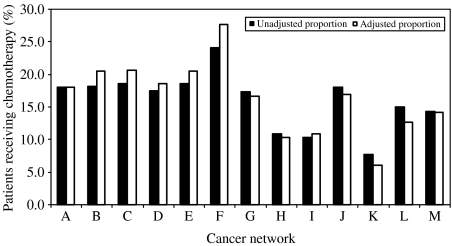
Percentage of patients receiving chemotherapy according to cancer network.

A significant difference was seen in the proportion of patients receiving chemotherapy in the different cancer networks ranging from 8 to 24% (Figure 4). For patients with NSCLC, the variation in chemotherapy use across the cancer networks was between 4.4 and 23.2%. For patients with SCLC, this variation across cancer networks was between 35.8 and 64.7% (data not shown). The overall lowest use of chemotherapy for both cancer types was network K. Representation of the cancer networks is shown by letters (A to M) to ensure anonymity.

There was an association between the stage of the cancer and use of chemotherapy. The highest proportion of chemotherapy use was in patients with stage III cancer, although there was no significant difference (adjusted proportion) between patients with stages II, III and IV receiving chemotherapy. However, after adjustment for other factors, stage II patients were more likely to be initiated with chemotherapy.

## DISCUSSION

Chemotherapy is a systemic form of cytotoxic therapy, indicated for both NSCLC and SCLC, and can be used in combination with radiotherapy and surgery. Radical surgery is only available for patients with early stage NSCLC, leaving palliative forms of treatment to manage and reduce symptoms, help improve quality of life and in some cases extend life expectancy (Jaklitsch *et al*, 2003; Todd, 2005). The choice of treatment is dependent on the type of cancer, health status and co-morbidity of the patient. The most important factors to consider regarding choosing chemotherapy are the side effect profiles of the medication, use of concurrent radiotherapy, performance status of the patient, patient and carrier preference, and total cost difference between the various chemotherapy regimens. When taking into account the performance status of the patient the stage, type of cancer, age and co-morbidity of the patient become important.

By far, the strongest determinant of use of chemotherapy appears to be age, and this effect is remarkably insensitive to statistical adjustment for type of cancer or stage. In general, the highest proportion of patients suffering from lung cancer are over the age of 65 (ONS, 2001; Spiro and Silvestre, 2005). The choice of management must be individually considered and should be based on the stage of the cancer, clinical and functional status, concomitant disease, nutritional status and cognitive function. The patient's age should not be a contraindication for the commencement of treatment. Shepherd *et al* (1994) came to a similar conclusion when they looked at chemotherapy in elderly patients in North America. However, it is appreciated in this study that age is associated with other co-morbid conditions, and performance status, which may contraindicate chemotherapy and it remains to be determined if the apparent age effect is independent of these factors. It is acknowledged that performance status is the most important factor in determining chemotherapy, and it is likely that most randomised trials have not included patients because of poor performance status. However, no information about performance status was available and, therefore, we can only speculate. It still remains a possibility that the use of chemotherapy in older patients is below the optimal level and that increased use of chemotherapy in these patients could increase survival and quality of life.

In this study, stage influenced the use of chemotherapy in NSCLC, with patients having stage II cancer being more likely to receive treatment compared to those with stage IV. In the case of stage IIIB or IV NSCLC, which is generally not considered curable, with 5-year survival rates of less than 1%, chemotherapy options are limited. However, more recently, newer drugs and regimens have been found to combat more aggressive forms of cancer, the importance of which is limited by our study design. It is also known that targeting therapy at an early stage of cancer increases the chance of eradicating the cancer and prolonging life. The fact that chemotherapy is introduced early may indicate improvements in lung cancer diagnosis and detection.

NICE recommends the routine use of chemotherapy in SCLC (NICE, 2001a). Chemotherapy in SCLC provides a more favourable outcome in patients with disease confined to the chest, minimal weight loss and good performance status. For patients with NSCLC, chemotherapy is recommended as adjuvant therapy and for the palliative treatment of advanced NSCLC. In this study, a greater proportion of patients with SCLC were shown to receive chemotherapy compared to patients with NSCLC and NOS. This result suggests some concordance with national guidelines, but the overall proportion of patients receiving chemotherapy remains low (only 60%) despite the aggressiveness of SCLC. A US study of limited disease SCLC found that approximately 70% of patients received chemotherapy or chemoradiotherapy (Gaspar *et al* 2005). Differences in the numbers of SCLC patients receiving chemotherapy in the UK compared to countries such as North America and Japan may reflect differences in the choice of drugs used, previous low success rates of treatment with older drugs and selection criteria based on performance. Also, the role of surgery continues to be explored, more in Europe than in North America or Asia (Turrisi and Sherman, 2002). A Swedish study found that 38% of patients did not receive lung cancer specific treatment (Myrdal *et al*, 2004). However, it is not uncommon for patients to reject the option of chemotherapy for advanced stages of lung cancer. It was estimated that in 2001, only 5–20% of patients received palliative chemotherapy (NICE, 2001b).

The proportion of patients receiving chemotherapy increased year-on-year from 1994 to 2003. For patients with NSCLC there has been a gradual increase in chemotherapy use, whereas for those patients with SCLC, where treatments have been established for many years, the overall use has not increased. Contributing to the overall increase in chemotherapy with time are better detection of lung cancer, increased effectiveness of chemotherapeutic regimens with lower side effect profiles, and use of chemotherapy in combined modalities, meaning more patients have chemotherapy indicated as part of their therapy. The greater acceleration seen in use of chemotherapy after the year 2000 may be related to the release of The NHS Cancer Plan in 2000, and subsequent release of NICE guidelines. However, the actual impact of the increase in cancer care and services since the introduction of the guidelines has yet to be determined.

Across England, cancer networks provide the operational model for cancer services. Each cancer network is responsible for the delivery of all cancer services within a geographical area. There are 34 cancer networks spread geographically throughout the country, and these were commissioned after the publication of the Calmine-Hime report in April 1995 (Munro, 2001). However, there is considerable variation between the cancer networks in their use of chemotherapy for lung cancer. Further analysis in the use of chemotherapy in patients with NSCLC and SCLC showed variations in use across the cancer networks (data not shown). It is interesting to note that variations in chemotherapy use in patients with SCLC across the cancer networks exist especially since this is a strong indication for chemotherapy and established treatments have existed for many years. For patients with NSCLC the variations in chemotherapy use across cancer networks, and with time, are large and as yet unexplained. Noteworthy is that the chance of having an approved treatment for lung cancer is not the same. For SCLC the difference is twofold and for NSCLC it is fivefold. This difference may reflect a reluctance in certain cancer networks towards offering treatment to a particular group of cancer patients. It would be interesting to extend this study to see if the same differences in chemotherapy use across networks over time were found in breast cancer, which has a longer tradition for chemotherapy use.

Additionally, variations between cancer networks may be driven by disparity in financial resources available for chemotherapeutic services or it may be due to variation in established or informal patterns of clinical practice in hospitals and cancer networks. Unfortunately, there are considerable differences with respect to data capture, availability, accuracy and completeness of the data from the individual networks. The variation between cancer networks may in part reflect artefactual differences in data availability, but this is unlikely to account for a large part of the observed variation. It may be that the low use of chemotherapy in some of the networks suggests that these networks may simply be slow in implementing new treatments. If this is the case, then the differences cannot be explained by differences in patients, but rather in the knowledge of the treating physicians. Also, in the particular case of cancer network I, the Thames Cancer Registry covers only a small part, and it is likely that we have missed a proportion of chemotherapy episodes which took place in the part of this network which is covered by the adjacent cancer registry. We are not aware of any other obvious sources of artefactual variation between the cancer networks that would importantly influence the interpretation of the data.

Socio-economic factors have been identified as an important determinant of access to cancer services, with the less affluent populations having poorer access to specialist services and treatments. This inequality is commonly referred to as the ‘postcode lottery’ (Coleman *et al*, 2004; Bungay, 2005). The NHS Cancer Plan has recognised this inequality and has proposed methods of reducing these inequalities by improving access for the poor to specialist services and more importantly providing a comprehensive programme of guidance setting national standards. However, Coleman *et al* (2004) have shown that the poverty gap in cancer survival has widened in England and Wales. There may be many reasons for this variation, including diet, smoking and other lifestyle habits among the poor, as well as access to appropriately trained health personnel and therapy. While our data suggest a statistically significant gradient in the direction of more frequent use of chemotherapy in the highest socio-economic groups, the absolute differences are very small in comparison with the variations between age-groups or between cancer networks. Interestingly, studies in the USA by Earle *et al* (2002) and Greenberg *et al* (1988a, 1988b) into the variation in use of palliative chemotherapy for advanced non-small-cell lung cancer showed no relationship with race, socio-economic class and geographic location once referral by an oncologist was made.

All the factors considered in this study contributed to a different extent to those patients who received chemotherapy or not. It is interesting to note the reports in the media profiling patients who do not receive chemotherapy as a result of cost. Cost of chemotherapy in lung cancer patients can be considerable, and if NICE guidelines are followed with use of new generation and more expensive drugs, the impact on whether patients receive chemotherapy may widen the gaps already seen in cancer treatment within the NHS. By many accounts, the inequalities seen in cancer care and choice of drugs are still present and it may yet be too early to determine if the Cancer Plan is fulfilling all its objectives. In the case of lung cancer, use of chemotherapy is improving but advancements are needed in education and funding as these will have an impact on choice of therapy. Additionally, observations in to the use of chemotherapy for other cancers and across different regions and countries warrants further consideration.

## Figures and Tables

**Table 1:** Number, unadjusted and adjusted proportions of patients receiving chemotherapy within 6 months of diagnosis, by various factors

	**No. of patients receiving CT**	**Total no. of patients**	**Unadjusted proportion (%)**	**Adjusted proportion (%)**
*Age group*				
0–40	471	1092	43.1	43.1
45	645	1566	41.2	38.3
50	1126	2987	37.7	33.2
55	1542	4819	32.0	26.6
60	1847	6996	26.4	20.9
65	2140	10 290	20.8	16.6
70	1901	13 203	14.4	10.9
75	1163	12 627	9.2	6.6
80–100	382	13 732	2.8	2.1
				
Test for trend			*χ*^*2*^=5289.6 (*P*<0.001)	*χ*^2^=4602.7 (*P*<0.001)
*Sex*				
Male	6958	41 667	16.7	16.7
Female	4257	25 645	16.6	15.4
				
Test for heterogeneity			*χ*^2^=0.12 (*P*=0.7309)	*χ*^2^=13.36 (*P*=0.003)
*IMD quintile*				
1 Least deprived	1734	9,999	18.3	18.3
2	2025	11 595	17.0	15.7
3	2486	13 099	15.9	14.5
4	2325	15 225	15.5	12.8
5 Most deprived	2646	17 394	17.4	12.8
				
Test for trend			*χ*^2^=6.51 (*P*=0.0107)	*χ*^2^=103.2 (*P*<0.001)
*Cancer type*				
NSCLC	4332	31 977	13.5	13.5
SCLC	4648	8668	53.6	60.7
NOS	2236	26 667	8.4	10.0
				
Test for heterogeneity			*χ*^2^=9519.1 (*P*<0.001)	*χ*^2^=7394.3 (*P*<0.001)
*Year of diagnosis*				
1994	830	6116	13.6	13.6
1995	832	6352	13.1	13.8
1996	939	6266	15.0	16.0
1997	968	6543	14.8	16.7
1998	1065	7010	15.2	17.8
1999	1115	6915	16.1	19.2
2000	1319	7305	18.1	22.6
2001	1330	7072	18.8	24.6
2002	1392	6994	19.9	26.6
2003	1426	6739	21.2	29.3
				
Test for trend			*χ*^2^=306.5 (*P*<0.001)	*χ*^2^=672.2 (*P*<0.001)
*Cancer network*				
A	1466	8163	18.0	18.0
B	1414	7755	18.2	20.5
C	1155	6208	18.6	20.7
D	1377	7895	17.4	18.6
E	725	3902	18.6	20.5
F	1405	5839	24.1	27.7
G	972	5591	17.4	16.6
H	641	5912	10.8	10.3
I	115	1110	10.4	10.9
J	511	2833	18.0	16.9
K	354	4560	7.8	6.1
L	73	486	15.0	12.6
M	1008	7058	14.3	14.2
				
Test for heterogeneity			*χ*^*2*^=762.7 (*P*<0.001)	*χ*^*2*^=927.5 (*P*<0.001)
*Stage*				
I	3173	21 620	14.7	14.7
II	642	3095	20.7	19.2
III	425	2031	20.9	15.5
IV	4448	21 668	20.5	15.7
Not known	2528	18 898	13.4	13.5
Test for trend			*χ*^*2*^=243.4 (*P*<0.001)	*χ*^*2*^=5.0 (*P*=0.0255)
